# Epicardial adipose tissue and metabolic syndrome: An update protocol for systematic review and meta-analysis

**DOI:** 10.1097/MD.0000000000010387

**Published:** 2018-04-20

**Authors:** Leonardo Roever, Elmiro Santos Resende, Angélica Lemos Debs Diniz, Nilson Penha-Silva, João Lucas O’Connell, Paulo Fernando Silva Gomes, Hugo Ribeiro Zanetti, Anaisa Silva Roerver-Borges, Fernando César Veloso, Fernanda Rodrigues de Souza, Poliana Rodrigues Alves Duarte, Thiago Montes Fidale, Antonio Casella-Filho, Paulo Magno Martins Dourado, Antonio Carlos Palandri Chagas, Sadeq Ali-Hasan-Al-Saegh, Paulo Eduardo Ocke Reis, Rogério de Melo Costa Pinto, Gustavo B. F. Oliveira, Álvaro Avezum, Mansueto Neto, André Durães, Rose Mary Ferreira Lisboa da Silva, Antonio José Grande, Celise Denardi, Renato Delascio Lopes, Nitesh Nerlekar, Shahab Alizadeh, Adrian V. Hernandez, Maria Inês da Rosa, Giuseppe Biondi-Zoccai

**Affiliations:** a Federal University of Uberlândia, Department of Clinical Research, Master Institute of Education President Antonio Carlos, IMEPAC, Araguari; b Department of Clinical Research, HCFMUSP - University of São Paulo Medical School, Department of Cardiology; c Faculty of Medicine ABC, Department of Cardiology Santo André; d Cardiovascular Research Center, Shahid Sadoughi University of Medical Sciences, Department of Cardiology, Yazd, Iran; e Department of Specialized and General Surgery, Fluminense Federal University, Rio de Janeiro; f Dante Pazzanese Institute of Cardiology, Department of Clinical Research, São Paulo; g Graduate Program in Medicine and Health, Department of Health and Sciences, Federal University of Bahia; h Federal University of Minas Gerais, Department of Cardiology, MG; i Federal University of Mato Grosso, Department of Medicine, MT; j FOP Unicamp, Department of Clinical Research, SP; k Division of Cardiology, Duke University Medical Center, Department of Clinical Research, Durham, NC; l Monash Cardiovascular Research Centre and MonashHeart, Department of Cardiology, Clayton, Victoria, Australia; m Department of Medicine, Tehran University of Medical Sciences; n University of Connecticut/Hartford Hospital Evidence-Based Practice Center, Hartford, Department of Comparative Effectiveness and Outcomes Research Health Outcomes, CT; o Laboratory of Epidemiology, University of Extremo Sul Catarinense, Criciúma, Brazil; p Department of Medico-Surgical Sciences and Biotechnologies, Sapienza University of Rome, Latina; q Department of AngioCardioNeurology, IRCCS Neuromed, Pozzilli, Italy.

**Keywords:** epicardial fat, metabolic syndrome, systematic review

## Abstract

**Background:**

The prevalence of metabolic syndrome (MetS) and MetS-related stroke is set to increase dramatically in coming decades. MetS is a complex disease that includes endothelial dysfunction, insulin resistance, diabetes, hypertension, ectopic obesity, and dyslipidaemia, and an increased risk of cardiovascular events. However, there are no systematic analyses, or well-conducted meta-analyses to evaluate the relationship between epicardial adipose tissue (EAT) and (MetS). The aim of this study is to examine this association of EAT with MetS in different ages and sex.

**Methods:**

The update systematic review, and meta-analysis will be conducted using published studies that will be identified from electronic databases (ie, PubMed, EMBASE, Web of Science, and Google Scholar. Studies that firstly, examined the association between EAT and MetS, secondly, focus on cohort, case-control, and cross-sectional studies, thirdly, were conducted among in adults aged between 40 and 70 years, fourth, provided sufficient data for calculating ORs or relative risk with a 95% CI, fifth, were published as original articles written in English or other languages, and sixth, have been published until January year 2018 will be included. Study selection, data collection, quality assessment, and statistical syntheses will be conducted based on discussions among investigators.

**Results:**

Ethics approval was not required for this study because it was based on published studies. The results and findings of this study will be submitted and published in a scientific peer-reviewed journal. This study will provide a high quality synthesis on the association of EAT and MetS.

**Conclusion:**

This systematic review will provide evidence to assess whether there is a strong association of EAT and MetS, and its components.

## 1 Background

The prevalence of metabolic syndrome (MetS) and MetS-related stroke is set to increase dramatically in coming decades. The MetS describes an association among endothelial dysfunction, insulin resistance, diabetes, hypertension, ectopic obesity, and dyslipidemia, and an increased risk of cardiovascular disease, and stroke.^[1–7]^

It is in large part the result of unbalanced diet, low socio-economic, and cultural level, stress, and sedentary lifestyle. The epicardial adipose tissue (EAT) can increase in the states of positive energy balance, when the free fatty acids in the blood are converted into triglycerides, and accumulated initially in adipocytes, and with this the concentration of triglycerides in the myocardium, and the disorders of glucose-insulin metabolism, the presence of chronic low-grade inflammation, and increased pro-inflammatory cytokine production by adipocytes are related to the MetS and the EAT dysfunction.^[7–11]^

This study aims to systematically, assess the association between EAT and MetS in adults aged between 34 and 70 years; and to provide a framework to further understand these factors in order to better target prevention strategies.

## 2 Objectives

The primary objective is to identify, and summarize the association with EAT, and with MetS in adults (34 – 70 years) in different ages and sex. The objective of this systematic review, and meta-analysis was to explore the association between EAT functionality, and the presence of MetS. The secondary aims were to evaluate whether increasing EAT is associated with MetS presence, and the strength of association with presence of MetS by EAT, measurement method in MetS might add additional risk factor, and predictor of CVD events, and mortality (CV-mortality, mortality, and all-cause mortality).

## 3 Methods/design

This systematic review of the literature will follow the Preferred Reporting Items for Systematic Reviews and Meta-Analyses (PRISMA) recommendations. The databases PubMed, Embase, Web of Science, Google scholar, and Cochrane were searched for articles. Our search will focus on cohort, case-control, and cross-sectional studies examining the association between very low EAT and MetS. Two reviewers will independently, screen articles, extract relevant data, and assess the quality of the studies.

MetS will be defined according to the unified definition of MetS based on a Joint Interim Statement of the International Diabetes Federation Task Force on Epidemiology and Prevention; National Heart, Lung, and Blood Institute; American Heart Association; World Heart Federation; International Atherosclerosis Society; and International Association for the Study of Obesity. We also consider the patients with the MetS diagnosis based on previous definitions, mainly based on the National Cholesterol Education Program's Adult Treatment Panel III. Individuals will be classified as having MetS if they have 3 or more of the followings from UHS Visit 1: elevated BP (systolic BP ≥130 mmHg or diastolic BP ≥85 mmHg); elevated TG (≥ 150 mg/dL); low HDL-C (men <40 mg/dL, women <50 mg/dL); impaired fasting glucose (>100 mg/dL); and elevated waist circumference (WC) (men ≥94 cm, women ≥80 cm). Diabetes will be defined as a fasting plasma glucose level ≥126 mg/dL. Impaired fasting glucose will be defined as a fasting plasma glucose level between 100 to 125 mg/dL.^[1–7]^

The study is registered with PROSPERO (CRD42018083456). This protocol conforms to the Preferred Reporting Items for Systematic Reviews and Meta-Analyses Protocols (PRISMA-P) guidelines.^[12,13]^

### 3.1 Systematic review registration

This protocol is registered in the PROSPERO registry of the University of York Reference number: (CRD42018083456).

## 4 Eligibility criteria

The PICOS strategy (population, intervention (changed to exposure for the purposes of this review of observational studies), comparator, outcome, study characteristics) was used to define the eligibility criteria for this study:

Inclusion criteria:

Participants (men and women) aged 18 years and above. We are interested in both primary, and secondary prevention studies, and so our inclusion criteria feature both participants free of CVD at baseline, and populations of patients admitted with MetS.

Exclusion criteria: Aged 0–18 years.

Pregnant women.

Data items on the following 5 domains will be extracted:

(1) *Population:* characteristics of the study population (eg, mean/median age, ethnic distribution), inclusion and exclusion criteria
(2) *Exposure:* definition and identification of EAT. Both criteria must be met:
  1. Measurement of EAT by linear, or volumetric analysis using MRI, CT, or echocardiography.
  2. Patients who have undergone a full 3D assessment of the myocardium in order to obtain full volumetric measurement of the EAT. This can only be performed with MRI or CT imaging. Echocardiography as an imaging modality to obtain myocardial function, specifically measures of systolic function (e.g. ejection fraction), diastolic function (e’, E/A ratio, and other doppler derived indices, as well as global longitudinal strain analysis), or full volume cardiac CT or MRI imaging to obtain ventricular volumes function parameters.
(3) *Comparators:* definition and identification of unexposed individuals, number of unexposed subjects
(4) *Outcomes:* definition and identification of primary CVD outcomes and death (CV mortality, all-cause mortality), and secondary outcomes (MetS diagnostic)
(5) *Study characteristics:* authors, publication year, setting/source of participants, design, methods of recruitment, and sampling, period of study, length of follow-up time (if relevant), aims, and objectives.

## 5 Outcomes

### 5.1.1 Primary outcomes

Patients with a diagnosis of MetS that present CVD outcomes, and death (CV mortality, all-cause mortality). The association of EAT with the presence of MetS.

### 5.1.2 Secondary outcomes

The association of EAT with MetS, by EAT measurement method.

For studies meeting the inclusion criteria, we will additionally assess the following secondary outcomes: TIA (a transient episode of neurological dysfunction caused by focal brain, spinal cord, or retinal ischaemia without acute infarction), and subtypes of stroke (ischaemic vs haemorrhagic). Most strokes (approximately 85%) are ischaemic (an episode of neurological dysfunction caused by focal, cerebral, spinal, or retinal infarction), compared with haemorrhagic (neurological dysfunction caused by a focal collection of blood within, or on the surface of the brain). Eligibility criteria may be further developed, in an iterative process, after preliminary searches.

### 5.2 Study design

**Figure 1:**
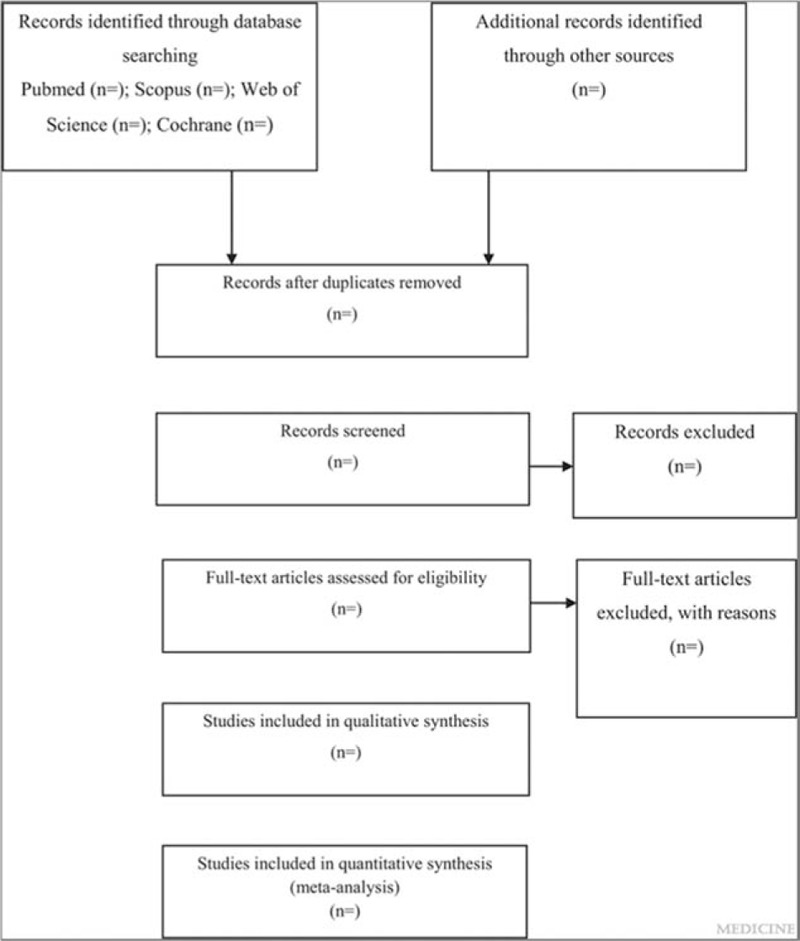
Flow diagram of study selection process.

This is a systematic review and meta-analysis protocol of prospective cohort studies, following the PRISMA-P (Preferred Reporting Items for Systematic Reviews and Meta-Analysis protocols) guideline.^[13]^ The systematic review and meta-analysis will be reported according to the PRISMA (Preferred Reporting Items for Systematic Reviews and Meta-Analyses) guideline.^[14]^ The whole process of study selection is summarized in the PRISMA flow diagram (Fig. 1).

### 5.3 Search strategy

A systematic review of the literature will be conducted. A language restriction shall not be applied to the search. If there are relevant non-English abstracts, attempts shall be made to translate them wherever possible.^[15]^ The following bibliographic databases (Embase, PubMed-MEDLINE, Web of Science, Cochrane Library, and Google Scholar) will be searched for articles published until January 2018.

Our search focuses on studies examining the association between EAT and MetS risk in adults (34–70 years).^[11]^ At each step of the selection process, reasons for inclusion/exclusion will be recorded in the PRISMA flowchart.^[12]^

### 5.4 Data collection

A record will be kept of all searches, and search decisions to ensure reproducibility. Search results will be exported to a citation management program (EndNote ver. 7.0; Clarivate Analytics, Philadelphia). Duplicates will be removed and retained separately. The resulting references will be exported separately, to the 2 reviewers for independent review using Covidence.

### 5.5 Selection of studies

Two authors (LR, FCV) will independently, screen all titles, and abstracts identified through the literature searches, and will exclude all records clearly, not meeting inclusion criteria. Disagreements will be resolved by consensus. The selection process will be pilot tested to ensure a high degree of agreement between reviewers. Full text of the remaining studies will then be retrieved. The same 2 authors (LR, FCV) will independently, assess the papers for fulfilment of inclusion criteria. In case of differences of opinion regarding study inclusion, a third review author (GBZ) will serve as arbiter. To avoid double counting, if multiple publications based on the same cohort of participants are retrieved, only the study reporting the largest sample size will be used. The reasons for excluding papers for which the full text was retrieved will be documented.

### 5.6 Data extraction and management

A data extraction form will be used to collect details from the included studies. The form includes information on study design, patient population, and presence of MetS. Two review authors (LR and FCV) will independently, extract the data. The data extraction form will be pilot tested on several papers to ensure consistency, and that all relevant information is being captured. If necessary, a statistician will review the extraction of data to further ensure quality and reliability. Authors will be contacted for missing data.

Data will be extracted using a standardized template. We will use the PICOS (Population, Intervention, Comparator, Outcomes and Study design) framework, originally, devised to formulate a research question, as a basis to develop data extraction criteria. As this is an aetiological study, “exposure” will replace “intervention” and “study characteristics” will replace “study design.”

In terms of the study results, unadjusted, and fully, adjusted effect estimates for the association between EAT and MetS will be recorded. Details of the confounders measured and adjusted for will also be noted. Results of any additional stratified analyses will also be recorded. Where possible, results from additional subgroup analyses with evidence regarding our non-primary objectives will also be recorded, for example, the association between EAT and the secondary outcomes.

### 5.7 Assessment of methodological quality

Two investigators (LR and FCV) will independently, assess each selected study for study quality using the Newcastle-Ottawa Quality Assessment Scale (NOS).^[16]^ The NOS evaluates cohort studies based on 8 items categorized into the following 3 groups: firstly, selection of the study cases, secondly, comparability of the population, and thirdly, ascertainment of whether the exposure, or outcome includes any risk of bias (i.e., selection bias or bias from lost to follow-up). The NOS is scored ranging between 0 and 9, and studies with scores ≥7 are considered as high quality.^[16]^ Discrepancy of quality assessment among the investigators will be solved by discussion, and consensus among all authors.

### 5.8 Data synthesis and statistical analysis

We anticipate that there may be significant heterogeneity in the prevalence of EAT features of MetS. There are several factors that could contribute to such heterogeneity. The relative risk (RR), and odds ratio (OR) are the way the result will be expressed statistically.

These factors include the following: differences in demographic, and clinical features (e.g., age, hypertension, renal disease, smoking, duration, and severity of diabetes) among study cohorts; differences in definitions of EAT. An I^2^ statistic will be calculated for the studies will be included in each proposed meta-analysis (i.e., for each neuroradiology correlate of interest) with values of 25, 50, and 75% suggesting low, moderate, or high degrees of heterogeneity, respectively, which report a dichotomized (i.e., present or absent), or categorical (i.e., absent, mild, moderate, severe), shall be harmonized for meta-analysis, if deemed appropriate by our statistician. Other types of rating scales shall not be included in a meta-analysis, and the data based on any such data scale would be presented in narrative form.

If significant heterogeneity between studies, as determined by consultation with our statistician, prevents meaningful pooling of the data, we will limit ourselves to provide a narrative description of observed trends. Given the heterogeneity of the populations studied, assumption of a fixed effect size across populations would not be justified, thus analyses would be performed using a random effects model. Given the dichotomized (presence or absence), or categorical (severity measure) nature of our data of, meta-analysis will be performed a random effects analysis. We will also add funnel graphs, publication bias analysis, and a meta-regression analysis.

If there are sufficient data to allow such analyses (in principle from as few as a single high-quality study, but if possible by pooling data from multiple studies), we will perform subgroup analyses for participants with renal disease, and participants with hypertension. Additionally, if sufficient data are available, we shall perform subgroup analyses by age, and diabetes duration. Funding sources and conflict of interest will be extracted from included studies. Statistical analysis will be performed using RevMan software; Review Manager (RevMan) [Computer program]. Version 5.3. Copenhagen, Denmark.

### 5.9 Strategy for data synthesis

The data of interest presented as continuous (mean value and SD) will be used to perform meta-analysis to obtain the standardized mean difference (SMD), and 95% confidence interval (CI). Cocharn's Q-statistic, and I-squared test will be used to test for heterogeneity between the included studies. If an I-squared value will be greater than 50%, or a *P* value of the Q-test will be less than 0.05, indicating maximal heterogeneity among the included studies, a random-effect model will be put into use. We will also add funnel graphs, publication bias analysis and a meta-regression analysis that were not included in previous meta-analyses.^[17]^

### 5.10 Analysis of subgroups or subsets

The subgroup meta-analyses will be conduct according to the pre-specified study-level characteristics using a fixed-effects meta-analysis, and if there is substantial heterogeneity, we will use the random effects model. The sources included location, sex, age, method of EAT assessment, the definition of MetS. We also will conduct sensitivity analyses to evaluate the potential sources of heterogeneity in the analyses.

### 5.11 Summary of Evidence

We will produce a narrative synthesis of the main results extracted from articles in full text. A summary of the included studies will provide information on the authors, study design, participants, number, and age of the subjects, theoretical structure (if relevant), alcohol consumption (as primary outcome of interest), main findings, study information. Special emphasis will be placed on the identification of EAT and the risk of MetS. In the presentation of the results, we will try to separate the factors for which the evidence of causality is strong (from longitudinal studies), and factors for which the causal nature of the relationship is less secure (cross-sectional data). A graphical summary of all the data they represent will be provided, and take into account the number of studies that provide evidence of a factor, and the relative strength of the association presented based on study design, and quality assessment. The membership level will be evaluated based on adjusted data.

## 6 Discussion

This systematic review will synthesize research evidence to establish whether the risk of developing MetS is relatively, high in adults with high EAT. Strengths and limitations will be highlighted in the identified evidence. Strength of observational data may include large sample size, high rate of follow-up, and frequency of METS more likely, to be representative of the population at risk. Limitations may include the quality of data extracted which may not allow studies to be combined in a meta-analysis. This may be overcome by presenting the findings in a descriptive manner. This review will be conducted in collaboration with an experienced librarian who helped appraise the search criteria, refine the keywords, and MeSH terms, and identify appropriate database(s). To the best of our knowledge, no reviews have been published exploring the study question; however, if a review addressing a similar question is published, it will be incorporated in this review, and added in a meta-analysis if feasible.

### 6.1 Implications of results

This systematic review will provide an updated, and quantifiable estimate of the risk of MetS in adults with high EAT. Furthermore, the systematic search will identify where future research is required. For instance, this review may inform a prognostic study which may be useful in understanding the course, and factors associated with MetS development.

### 6.2 Strengths and limitations of this study

This systematic review and meta-analysis will offer better understanding regarding the association between EAT and MetS.

The findings from this study will be useful for assessing of EAT and the risk factors in MetS, and determining approaches for prevention of MetS the future.

An improved understanding of this relationship may help to inform public health MetS prevention strategies.

Included studies may have substantially differ methodologies, which could limit our ability to draw reliable conclusions from the existing evidence base. Depending on the results, confounding factors that were not adjusted for in the selected studies, and low generalizability can be limitations. Individual patient data will not be available.

## Author contributions

**Conceptualization:** Leonardo Roever, Elmiro Santos Resende, Angélica Lemos Debs Diniz, Nilson Penha-Silva, João Lucas O’Connell, Paulo Fernando Silva Gomes, Hugo Ribeiro Zanetti, Anaisa Silva Roerver-Borges, Fernando César Veloso, Fernanda Rodrigues de Souza, Poliana Rodrigues Alves Duarte, Thiago Montes Fidale, Antonio Casella-Filho, Paulo Magno Martins Dourado, Antonio Carlos Palandri Chagas, Sadeq Ali-Hasan-Al-Saegh, Paulo Eduardo Ocke Reis, Rogério de Melo Costa Pinto, Gustavo B. F. Oliveira, Alvaro Avezum, Mansueto Neto, André Durães, Rose Mary Ferreira Lisboa da Silva, Antonio José Grande, Celise Denardi, Renato Delascio Lopes, Nitesh Nerlekar, Shahab Alizadeh, Adrian V. Hernadez, Maria Inês da Rosa, Giuseppe Biondi-Zoccai.

**Methodology:** Leonardo Roever.

**Writing – original draft:** Leonardo Roever, Elmiro Santos Resende, Angélica Lemos Debs Diniz, Nilson Penha-Silva, João Lucas O’Connell, Paulo Fernando Silva Gomes, Hugo Ribeiro Zanetti, Anaisa Silva Roerver-Borges, Fernando César Veloso, Fernanda Rodrigues de Souza, Poliana Rodrigues Alves Duarte, Thiago Montes Fidale, Antonio Casella-Filho, Paulo Magno Martins Dourado, Antonio Carlos Palandri Chagas, Sadeq Ali-Hasan-Al-Saegh, Paulo Eduardo Ocke Reis, Rogério de Melo Costa Pinto, Gustavo B. F. Oliveira, Alvaro Avezum, Mansueto Neto, André Durães, Rose Mary Ferreira Lisboa da Silva, Antonio José Grande, Celise Denardi, Renato Delascio Lopes, Nitesh Nerlekar, Shahab Alizadeh, Adrian V. Hernadez, Maria Inês da Rosa, Giuseppe Biondi-Zoccai.

**Writing – review & editing:** Leonardo Roever, Elmiro Santos Resende, Angélica Lemos Debs Diniz, Nilson Penha-Silva, João Lucas O’Connell, Paulo Fernando Silva Gomes, Hugo Ribeiro Zanetti, Anaisa Silva Roerver-Borges, Fernando César Veloso, Fernanda Rodrigues de Souza, Poliana Rodrigues Alves Duarte, Thiago Montes Fidale, Antonio Casella-Filho, Paulo Magno Martins Dourado, Antonio Carlos Palandri Chagas, Sadeq Ali-Hasan-Al-Saegh, Paulo Eduardo Ocke Reis, Rogério de Melo Costa Pinto, Gustavo B. F. Oliveira, Alvaro Avezum, Mansueto Neto, André Durães, Rose Mary Ferreira Lisboa da Silva, Antonio José Grande, Celise Denardi, Renato Delascio Lopes, Nitesh Nerlekar, Shahab Alizadeh, Adrian V. Hernadez, Maria Inês da Rosa, Giuseppe Biondi-Zoccai.
